# Inhibition of orthotopic castration-resistant prostate cancer growth and metastasis in mice by JC VLPs carrying a suicide gene driven by the PSA promoter

**DOI:** 10.1038/s41417-023-00699-8

**Published:** 2023-12-11

**Authors:** Chih-Chieh Chou, Chih-En Tseng, Yu-Shih Lin, Meilin Wang, Pei-lain Chen, Deching Chang, Cheng-Huang Shen, Chiung-Yao Fang

**Affiliations:** 1 Institute of Molecular Biology, National Chung Cheng University, Chiayi, Taiwan; 2 Department of Anatomic Pathology, Dalin Tzu Chi Hospital, Buddhist Tzu Chi Medical Foundation, Chia-Yi, Taiwan; 3 Department of Pharmacy, Chiayi Chang Gung Memorial hospital, Chiayi Branch, Puzi Taiwan; 4 Department of Microbiology and Immunology, School of Medicine, Chung-Shan. Medical University and Clinical Laboratory, Chung-Shan Medical University Hospital, Taichung, Taiwan; 5 Department of Medical Laboratory Science and Biotechnology, Central Taiwan University of Science and Technology, Taichung, Taiwan; 6 Department of Urology, Ditmanson Medical Foundation Chiayi Christian Hospital, Chiayi, Taiwan; 7 Department of Medical Research, Ditmanson Medical Foundation Chiayi Christian Hospital, Chiayi, Taiwan

**Keywords:** Cancer, Prostate cancer

## Abstract

Metastatic castration-resistant prostate cancer (mCRPC) is challenging to treat. Virus-like particles (VLPs), originating from JC polyomavirus (JCPyV) and carrying a suicide gene driven by the PSA promoter (PSAtk-VLPs), can inhibit tumor growth in animal models of human prostate cancer. However, the efficacy of suppression of orthotopic PCa growth and metastasis by PSAtk-VLPs remains undetermined. Here, we established an iRFP stable expression CRPC cell line suitable for deep-tissue observation using fluorescence molecular tomography (FMT). These cells were implanted into murine prostate tissue, and PSAtk-VLPs were systemically administered via the tail vein along with the prodrug ganciclovir (GCV), allowing for the real-time observation of orthotopic prostate tumor growth and CRPC tumor metastasis. Our findings demonstrated that systemic PSAtk-VLPs administration with GCV and subsequent FMT scanning facilitated real-time observation of the suppressed growth in mouse iRFP CRPC orthotopic tumors, which further revealed a notable metastasis rate reduction. Systemic PSAtk-VLPs and GCV administration effectively inhibited orthotopic prostate cancer growth and metastasis. These findings suggest the potential of JCPyV VLPs as a promising vector for mCRPC gene therapy. Conclusively, systemically administered JCPyV VLPs carrying a tissue-specific promoter, JCPyV VLPs can protect genes within the bloodstream to be specifically expressed in specific organs.

## Introduction

Prostate cancer (PCa) is the fifth leading cause of cancer-related deaths and the second most frequently diagnosed cancer among men, making PCa therapy a pressing health issue worldwide [1, 2]. Surgical resection or radiation therapy is typically the initial treatment for localized PCa [3]. However, despite these measures, the disease continues to progress in some patients, and androgen deprivation therapy (ADT) is then used as a treatment for advanced PCa [4, 5]. However, after ADT treatment, certain patients eventually develop castration-resistant prostate cancer (CRPC) [6]. Despite novel drug development for CRPC treatment, drug resistance remains inevitable. Nearly all patients ultimately progress to lethal metastatic castration-resistant prostate cancer (mCRPC) [7, 8], establishing the current challenge in PCa treatment.

Androgens and AR signaling are pivotal in mCRPC progression, and drugs have been developed to target the AR pathway [9, 10]. Abiraterone binds to an important androgen-synthesizing enzyme, cytochrome P 17, which in turn inhibits androgen synthesis [11]. The AR antagonist, Enzalutamide, directly binds to AR, inhibiting processes such as androgen binding to AR, AR nuclear translocation, and DNA binding [12]. New-generation drugs, such as apalutamide and darolutamide, increase the survival rate of mCRPC patients [13]. However, aberrations found in AR, including gene amplification, overexpression, rearrangements, splice variants, and activating mutations lead to drug resistance in patients [14]. If PCa patients have mutations in DNA repair genes, such as BRCA 1/2, the non-AR related drug poly (ADP-ribose) polymerase (PARP) inhibitor can be used; however, it only slightly prolongs the survival of patients with mCRPC [15] and the issue of drug resistance persists [16]. Therefore, mCRPC remains a lethal disease in men, highlighting the urgent need for novel treatment strategies.

Considering the drug resistance issues of current PCa treatments, gene therapy for PCa treatment is currently under development [17]. A major hurdle is systemic delivery and metastatic PCa targeting [18]. Prostate-specific antigen (PSA) expression is regulated by androgens and AR signaling and is expressed in normal and prostate tumor tissues. Furthermore, the expression increases with PCa progression. Therefore, using the PSA promoter to drive therapeutic gene expression is an effective strategy for specific PCa treatment [19, 20]. Previously, we constructed a thymidine kinase (tk) suicide gene expression plasmid (pPSAtk) regulated by a PSA promoter. In the presence of the prodrug, GCV, it effectively eliminates AR-positive CRPC cells. Human JC polyomavirus virus-like particles (JCPyV VLPs) encapsulating pPSAtk inhibit human prostate cancer growth in murine models without affecting non-prostate tissues [19, 21, 22]. In the orthotopic mouse xenograft model, tumor cells were implanted into murine organs corresponding to the origin of cancer in humans to represent growth in a similar environment to the original tumor, mimicking human tumor development. This model can provide valuable information for preclinical research [23, 24]. In this study, we built upon our previous findings to further establish an animal model for the real-time observation of human mCRPC orthotopic prostate cancer. Using fluorescence molecular tomography (FMT), which is suitable for deep tissue observation, mCRPC cells carrying near-infrared fluorescent protein (iRFP) were implanted into murine prostate tissue. With systemic PSAtk-VLPs and GCV administration, we regularly monitored the intensity of iRFP fluorescence (representing the tumor) which allowed us to evaluate the ability of PSAtk-VLPs to inhibit mCRPC orthotopic tumor growth and prevent mCRPC from metastasizing to other organs.

## Materials and methods

### Cell lines

The human prostate cancer cell line, 22Rv1, was procured from The Bioresource Collection and Research Center (Hsinchu, Taiwan). The 22Rv1 cells were cultured in RPMI-1640 media containing L-glutamine, sodium pyruvate acid, and HEPES supplemented with 10% fetal bovine serum (FBS) and a 1% penicillin/streptomycin antibiotic solution (all from Gibco; Thermo Fisher Scientific, Cambridge, MA). The cell lines were incubated in a humidified atmosphere containing 5% CO_2_ at 37 °C. Cell lines were tested for mycoplasma contamination monthly.

### JCPyV VLP preparation

The preparation of JCPyV VLPs and the method of encapsulating genes are described in previous studies [21, 22, 25, 26]. Either the GFP expression plasmid, pEGFP-N3 (Clontech, Mountain View, CA), or the pPSAtk plasmid [21, 22] were co-transformed into *Escherichia coli* JM109 (Promega, Madison, WI) with the JCPyV VP1 expression plasmid, △pFlag-VP1. The co-transformed JM109 were cultured in Luria-broth (LB) culture medium containing kanamycin (30 μg/ml) and ampicillin (100 μg/ml) antibiotics to select for *E. coli* simultaneously containing pEGFP-N3 or pPSAtk and △pFlag-VP1. JCPyV VP1 protein expression was induced by adding 0.5 mM (final concentration) isopropyl β-D-1-thiogalactopyranoside (IPTG) for 16 h at 30 °C. After induction, the *E. coli* were lysed, supernatant was collected, and then JCPyV VLP encapsulating pEGFP-N3 or pPSAtk was purified by 10–30% sucrose gradient centrifugation at 30000 r.p.m for 2.5 h at 4 °C using a Beckman SW-41 Ti rotor in a Beckman L8-70M ultracentrifuge. The plasmid-encapsulated JCPyV VLP was then concentrated using a centricon filter (Millipore, Billerica, Ma, USA) and stored in Tris-buffered saline (10 mM Tris–HCl, pH 7.4, 150 mM NaCl) at –80 °C for subsequent experiments.

### Establishment of 22Rv1CRPC cell line expressing iRFP-tGFP fluorescent protein

piRFP was a gift from Vladislav Verkhusha (Addgene plasmid # 31857) [27]. Using lentiviral vectors (National RNAi Core Facility, Taiwan) carrying the coding sequences of iRFP (piRFP; Addgene, USA) and GFP (pLKO_AS3w. tGFP; RNAi Core Lab, Taiwan), we transfected the 22Rv1 cell line and analyzed the ratio of cells expressing iRFP and GFP fluorescence using flow cytometry (FACSAccuri™ C6 Plus; BD Biosciences). High-speed cell sorting (FACSAriaIII; BD Biosciences) was then used to select for 22Rv1 cells expressing iRFP at an excitation wavelength of 710 nm; these cells were then termed “22Rv1-iRFP-tGFP.” We then observed the fluorescence expression of iRFP and GFP in 22Rv1-iRFP-tGFP via confocal microscopy (Carl Zeiss, Thornwood, NY). 22Rv1-iRFP-tGFP cells of 5 × 10^5^ and 1 × 10^6^ were placed in the agent calibration phantom of FMT to detect whether the iRFP fluorescence signal representing the cells increased with the number of cells. The culturing method for 22Rv1-iRFP-tGFP was the same as for the parental 22Rv1 cells.

### Human orthotopic prostate cancer animal model establishment and JCPyV VLP gene delivery ability assessment

Animals were housed and raised in special pathogen-free (SPF) airflow cabinets. The animal care and treatment were performed according to the guidelines for experimental animals from the Institutional Animal Care and Use Committee of National Chung Cheng University (IACUC Approval No. 1100705). All animal procedures were performed according to approved protocols and in compliance with the recommendations in ARRIVE guidelines [28]. Six 6-week-old male nude mice (CAnN.Cg-Foxn1^nu^/CrlNarl, National Laboratory Animal Center, Taipei, Taiwan) were used to establish the human orthotopic prostate cancer animal model as per previous reports [29, 30]. During surgery, the male mice were anesthetized with 2.5% isofluorane, and the lower abdominal region was disinfected with povidone-iodine scrub (Betadine, Sinphar Pharmaceutical Co., Yilan, Taiwan) and alcohol pads (Dukal Corporation, Syosset, NY, USA). A low midline abdominal incision of approximately 3–4 mm was made where the left seminal vesicle was located, pulled out, and gently placed on a sterile gauze moistened with PBS. Using forceps and a 27-gauge syringe, we gently secured the left seminal vesicle and implanted 1 × 10^6^ 22Rv1 or 1 × 10^5^ 22Rv1 in 50 µL HBSS (Thermo Fisher Scientific, Cambridge, MA) into the anterior prostate lobe. Successful inoculation was confirmed by visible swelling of the murine prostate. The mice were randomly assigned into two groups, three in each group one week after the surgery. Each mouse was intravenously injected (IV) via the tail vein every other day with either VLPs (70 µg) or PSAgfp-VLPs (70 µg), for a total of six times. The mice were euthanized on day 22 post-tumor inoculation, and the orthotopic prostate tumors were removed. These tumors were then embedded in optimum cutting temperature (OCT) compound (Sakura Finetek, Torrance, CA), frozen, cut into sections of 7 µm thickness, and examined for GFP expression in the orthotopic prostate tumors using a fluorescence microscope (Carl Zeiss).

### Fluorescence molecular tomography (FMT) imaging

FMT (FMT 4000 MSIM; PerkinElmer, USA) was used to detect the overall iRFP fluorescence intensity in mice weekly for a total duration of 8 weeks. After weighing the mice, they were anesthetized with a mixture of isoflurane/oxygen and placed comfortably in the portable animal imaging cassette. The setting was established for a full-body scan, with excitation at the wavelength of 680 nm, initiating a 3D scan to quantify iRFP intensity. The scan covered the entire murine body with an excitation power set to 80 mW and exposure time between 2 and 5 s. Each mouse was scanned for 5–10 min using source-detector pair excitation measurements (λ_ex_ = 670 nm/λ_em_ = 690–740 nm) to quantify fluorescence signal intensity. The collected fluorescence signal data were reconstructed using TrueQuant v3.0 software (PerkinElmer), and the fluorescence data from a known concentration of the imaging agent, AngioSense 680 (PerkinElmer), was used to establish a standard curve to calibrate the iRFP fluorescence picomolar data representing the size of human prostate cancer tumors in mice.

### FMT imaging for real-time analysis of the therapeutic effect in the orthotopic prostate cancer murine model

Twelve 6-week-old male nude mice (CAnN.Cg-Foxn1nu/CrlNarl, National Laboratory Animal Center, Taipei, Taiwan) were implanted with 5 × 10^5^ 22Rv1-iRFP-tGFP prostate cancer cells in 50 μL HBSS (Thermo) in the anterior prostate lobe. A week later, the mice were randomly divided into three groups, with four mice in each group: the first group was the control group that received no treatment; the second group was the VLP control group, which received 100 µg of VLPs via intravenous (IV) tail injection every three days, followed by an intraperitoneal (IP) injection of 80 mg/kg ganciclovir (GCV) the next day for a total of seven cycles; the third group was the treatment group with JCPV VLP carrying the pPSAtk gene (PSAtk-VLPs), receiving 100 µg of PSAtk-VLPs via IV tail injection every three days, followed by an IP injection of 80 mg/kg GCV the following day for a total of seven cycles. On the day of random group assignment, the overall iRFP fluorescence intensity in each group of mice was detected using in vivo FMT (PerkinElmer) to establish baseline and background values before treatment. The iRFP fluorescence intensity was detected by FMT (PerkinElmer) weekly to reflect the real-time tumor size and the metastasis situation of orthotopic cancer cells for a total of eight weeks. At the end of the experiment, the mice were euthanized, and the orthotopic prostate tumor, lung, liver, spleen, kidney, digestive system, and femur were removed. The iRFP fluorescence intensity in each organ was directly detected using FMT to confirm the metastatic situation of prostate cancer cells. Furthermore, immunohistochemistry (IHC) was used to verify and compare the androgen receptor (AR) representing prostate cancer to the FMT-detected iRFP fluorescence intensity.

### Immunohistochemistry (IHC)

IHC was performed using a Histofine mouse stain kit (Nichirei, Tokyo, Japan) to detect AR and PSA expression and confirm the tumor origin of the 22Rv1 CRPC prostate cancer cells. Ki-67 marker was used to confirm cell proliferation. After the experiment, the murine organs were removed and fixed in 10% neutral buffered formalin (Sigma-Aldrich, St. Louis, MO, USA). The femur was decalcified for 72 h using Decalcifier II (Leica Biosystems, Buffalo Grove, IL). The tissue samples were then embedded in paraffin (Sigma–Aldrich) and cut into 5 μm tissue sections. The sections were deparaffinized thrice with Xylene (Muto pure Chemicals Co., Tokyo, Japan) for 30 min each and then underwent serial alcohol concentration dilution before being equilibrated in a Tris-buffered saline (TBS; 0.1 M Tris–HCl, pH 7.4, and 0.15 M NaCl) system. Heat-induced antigen retrieval (HIAR) was performed using an ethylenediaminetetraacetic acid (Tris-EDTA) buffer (pH 9.0), and the sections were incubated in 3% H_2_O_2_ for 10 min to quench endogenous peroxidase. The sections were then incubated with Blocking reagent A (Nichirei) for 1 h and then incubated with a primary antibody at 4 °C overnight. After washing, the sections were incubated with Blocking reagent B (Nichirei) for 10 min, followed by incubation with Simple Stain Mouse MAX PO (Nichirei) for 10 min at 37 °C. The sections were finally stained with a diaminobenzidine (DAB) (Sigma–Aldrich) substrate and counterstained with hematoxylin. Hematoxylin and eosin (H&E) staining was used to observe the histological structure and location of the prostate tumor. The primary antibody for AR was purchased from Santa Cruz (cat. No. sc-7305, Santa Cruz, California, USA). The PSA antibody was from Cell Margue (cat. No. EP-109, Cell Marque, Rocklin, CA). The Ki-67 antibody was from Thermo Fisher Scientific (RM-9106S, Thermo Fisher Scientific, Cambridge, MA).

### Statistical analysis

The fluorescent signal units quantified by FMT imaging as picomoles, relative whole-body fluorescent signals of the mice, weight of the mice, and fluorescent signals from the organs of the mice were evaluated using the One-way ANOVA Bartlett’s test to determine whether variances were similar in the available samples. The fluorescent signals of the tumor were tested using the one-way ANOVA unpaired t-test for comparison among three or more independent groups. *P* < 0.05 was considered statistically significant.

## Results

### JCPyV VLP successfully delivers genes to human prostate orthotopic tumors in mice

**Fig. 1:**
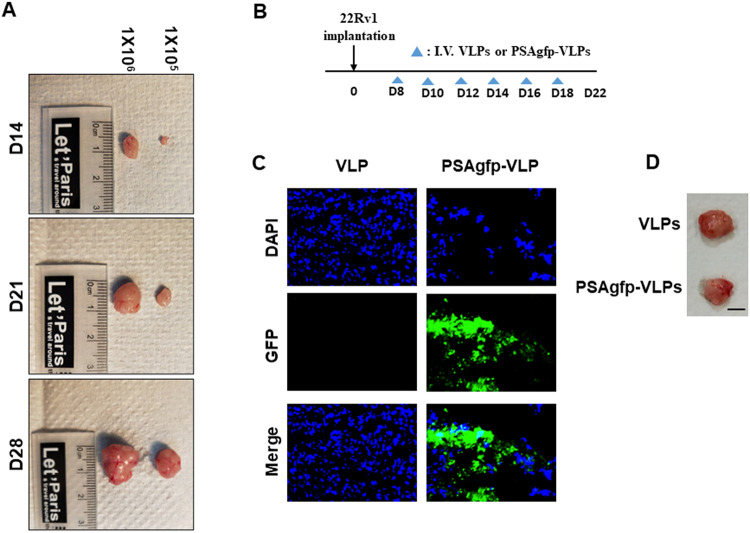
Green fluorescent protein (GFP) gene expression carried by JCPyV virus-like particles (VLPs) in human orthotopic prostate tumors in mice. **A** Murine model establishment of a human orthotopic prostate tumor. In human castration-resistant prostate cancer (CRPC), 22Rv1 cells were implanted into the anterior prostate lobe 6-week-old mice at densities of 1 × 10^5^ or 1 × 10^6^. The mice were euthanized on days 14, 21, and 28 to observe the orthotopic tumor sizes. **B** The administration process of PSAgfp-VLPs or VLPs. Eight days after 22Rv1 was implanted into the mouse prostate, PSAgfp-VLPs or VLPs were administered every two days via tail vein injection for a total of six times. **C** 22Rv1 was implanted into mice prostates and PSAgfp-VLPs or VLPs were administered. After 22 days, the orthotopic prostate tumor was removed, frozen sections were made, and GFP expression was observed under a fluorescent microscope. **D** The size of the orthotopic prostate tumor in mice with 22Rv1 cells after administration of PSAGFP-VLPs or VLPs. (Scale bar = 50 mm).

In an orthotopic mouse xenograft model, tumor cells can be implanted into murine organs (similar to the origin of cancer) for growth in a microenvironment equivalent to that of tumor occurrence, providing appropriate translation and information to clinics [23, 24]. Extending our previous experiment, where JCPyV VLP carried a suicide gene to inhibit the growth of human prostate cancer in a xenograft model [21, 22], in this study, we established a human prostate orthotopic cancer animal model to further evaluate the preclinical study of JCPyV VLP as a gene therapy vector for prostate cancer. The orthotopic tumor inoculation was slightly modified based on previous publications [29, 30]. Different numbers of CRPC 22Rv1 cells were implanted into the anterior prostate lobe of 6-week-old mice to evaluate the optimal cell number for establishing the orthotopic xenograft model, and the mice were euthanized in the second, third, and fourth weeks to observe the orthotopic tumor sizes. As the cell numbers and growth weeks increased, the tumor volume also increased (Fig. 1A). These findings demonstrate that we successfully established an orthotopic mouse xenograft model of human CRPC 22Rv1 cells. Next, we evaluated whether JCPyV VLP could carry and protect genes to human CRPC orthotopic mouse xenografts via systematic blood circulation. Eight days after 22Rv1 inoculation into the murine prostate, the mice were administered PSAgfp-VLPs or VLP via the tail vein every two days for a total of six times (Fig. 1B). The orthotopic prostate tumors were removed, freeze-sectioned, and the GFP expression was observed under a fluorescent microscope. Green fluorescence only appeared in the orthotopic prostate tumors of mice injected with PSAgfp-VLPs; whereas the group without any carried gene VLP did not exhibit green fluorescence (Fig. 1C). The tumor volume showed no significant differences between the PSAgfp-VLPs and VLP injection groups (Fig. 1D). These results indicate that JCPyV VLP can successfully carry and protect genes for expression in murine prostate orthotopic tumors via blood circulation. PSAgfp-VLPs did not affect tumor growth compared with the VLP control group.

### 22Rv1 CRPC cell line establishment stably expressed iRFP and GFP

**Fig. 2:**
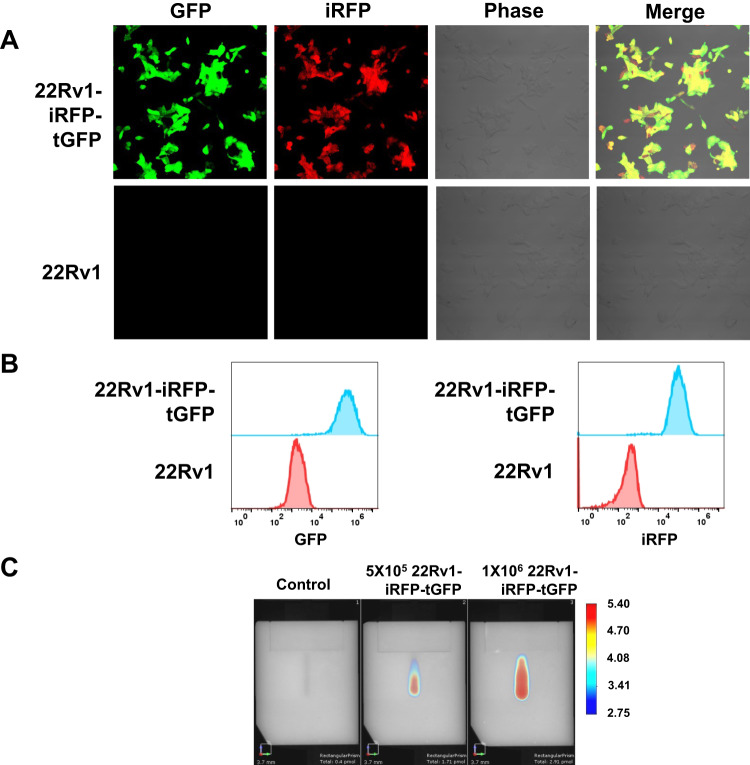
Stable near-infrared fluorescent protein (iRFP) and green fluorescent protein (GFP) expression in 22Rv1-iRFP-tGFP cells. **A** Confocal microscopic images for the GFP and iRFP fluorescence of 22Rv1-iRFP-tGFP and 22Rv1 cells. Scale bar = 100 μm. **B** Flow cytometry analysis of the GFP and iRFP expression ratio in 22Rv1-iRFP-tGFP cells. **C** iRFP fluorescence intensity detection with fluorescence molecular tomography (FMT) of 5 × 10^5^ and 1 × 10^6^ 22Rv1-iRFP-tGFP cells.

Fluorescence tomography techniques, in conjunction with a near infrared fluorescent protein (iRFP), provide deep tumor localization and quantitative tracking in mice [31]. Previously, we used human GBM cells stably expressing iRFP to track and record the size of intracranial GBM tumors in mice during gene therapy [32]. Using this technique, we aimed to track and record the treatment of orthotopic prostate tumors in mice in real-time. Firstly, we established CRPC cells, 22Rv1, which can stably express the reporter gene. By transfecting 22Rv1 cells with lentiviral vectors carrying GFP and iRFP, we obtained the resultant cells which were termed “22Rv1-iRFP-GFP.” We evaluated whether the 22Rv1-iRFP-GFP cells could stably express GFP and iRFP. The expression of GFP and iRFP fluorescent proteins in 22Rv1-iRFP-GFP cells was observed using confocal microscopy (Fig. 2A). Furthermore, the 22Rv1-iRFP-GFP cells were analyzed using flow cytometry to confirm GFP and iRFP fluorescent protein expression (Fig. 2B). Finally, we observed that fluorescence intensity increased with the increase in cell numbers using FMT (Fig. 2C). We also detected the PSA and Ki-67 expression by IHC to support the tumors originated from 22Rv1-iRFP-GFP cells and growth (Suppl. Fig. 2). Conclusively, we successfully established human CRPC cells, 22Rv1-iRFP-GFP, that stably express the reporter gene and we can measure their fluorescence intensity using FMT.

### FMT evaluated the efficacy of systemic PSAtk-VLP/GCV administration for human orthotopic prostate cancer treatment

After demonstrating that JCPyV VLP could carry the GFP gene to express in the 22Rv1 orthotopic prostate tumors in mice and that FMT can determine the fluorescence intensity of 22Rv1-iRFP-GFP cells, we further examined the inhibitory capability of JCPyV VLP (carrying the tk suicide gene) on the growth of orthotopic prostate tumors in the presence of GCV. We implanted 5 × 10^5^ 22Rv1-iRFP-GFP cells into the anterior prostate lobe in mice, and a week later, the mice were randomly assigned into three groups, namely the mock, VLP/GCV, and PSAtk-VLPs/GCV groups. The mock group received no treatment. The VLP/GCV group received an intravenous injection of 100 µg VLPs every three days, followed by an intraperitoneal (IP) injection of 80 mg/kg GCV the next day, forming one cycle of treatment which was repeated seven times. The PSAtk-VLPs/GCV group received an intravenous injection of 100 µg PSAtk-VLPs every three days, followed by an intraperitoneal (IP) injection of 80 mg/kg GCV the next day, for one cycle of treatment which was repeated seven times. We determined the iRFP fluorescence intensity representing tumor size with FMT weekly.

**Fig. 3:**
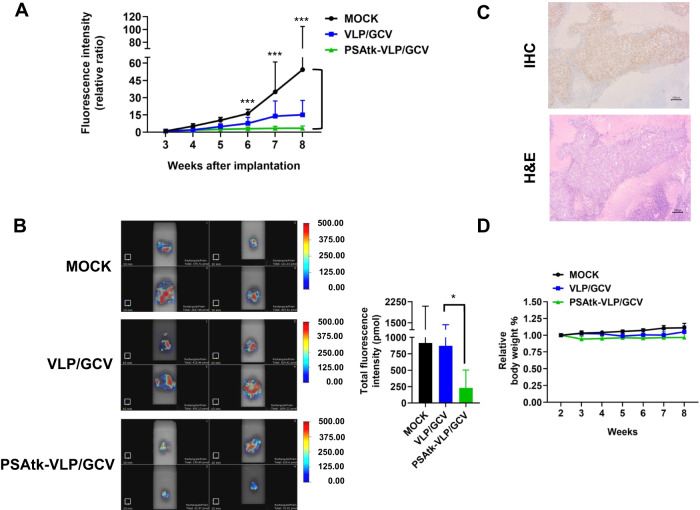
Fluorescence molecular tomography (FMT) imaging tracking the size of human castration-resistant prostate cancer (CRPC) orthotopic prostate cancer cells following PSAtk-VLP/GCV treatment. 22Rv1-iRFP-GFP cells were implanted into the anterior lobe of the murine prostate and divided into three groups. The mock group received no treatment. The VLP/GCV and PSAtk-VLPs/GCV groups received seven cycles of treatment with virus-like particles (VLPs) and ganciclovir (GCV) or PSAtk-VLPs and GCV, respectively. **A** Fluorescence intensity representing tumor size of the three groups of mice was measured weekly with in vivo FMT. Data are represented as means ± standard deviation (SD) *n* = 4, One-way ANOVA, Bartlett’s test, ***p < 0.005. **B** After different treatments, the mice were euthanized at week 8, and the orthotopic prostate tumors were removed. The fluorescence intensity (left) and quantitative image (right) of the tumor were obtained by ex vivo FMT imaging. *n* = 4, One-way ANOVA, Unpaired t test, *p < 0.05. **C** Immunohistochemistry (IHC) detection of androgen receptor (AR) expression and hematoxylin and eosin (H&E) staining in orthotopic prostate tumors. Scale bar = 100 μm. **D** Weekly measurements of the mice weight. Data are represented as mean ± standard deviation (SD), *n* = 4. One-way ANOVA, Bartlett’s test, *p* = 0.1305.

Two weeks following treatment initiation, each group exhibited detectable fluorescence signals in FMT (Suppl. Fig. 1, white arrow). As the treatment time increased, the group receiving JCPyV VLP (encapsulating the tk suicide gene) and the GCV injection (PSAtk-VLPs/GCV) showed a slowing trend in tumor volume increase; by contrast, the tumor volumes in the VLP/GCV and mock groups gradually increased (Fig. 3A, and Suppl. Fig. 1). At the end of the eighth week of the experiment, we removed the orthotopic prostate cancer tumors from the mice in each group and used FMT to detect and quantify the iRFP fluorescence signals representing the tumors. The average fluorescence intensity of the PSAtk-VLPs/GCV group was approximately one-third that of the control group (Fig. 3B). The tumor tissue expressed AR (Fig. 3C), and there were no significant differences in murine body weight between the groups (Fig. 3D). These results indicated that FMT could continuously monitor the growth of 22Rv1-iRFP-GFP orthotopic tumors, and that the tk gene driven by the PSA promoter (encapsulated by JCPyV VLP) could inhibit orthotopic prostate cancer growth.

### PSAtk-VLP/GCV inhibited orthotopic prostate cancer systemic metastasis

**Fig. 4:**
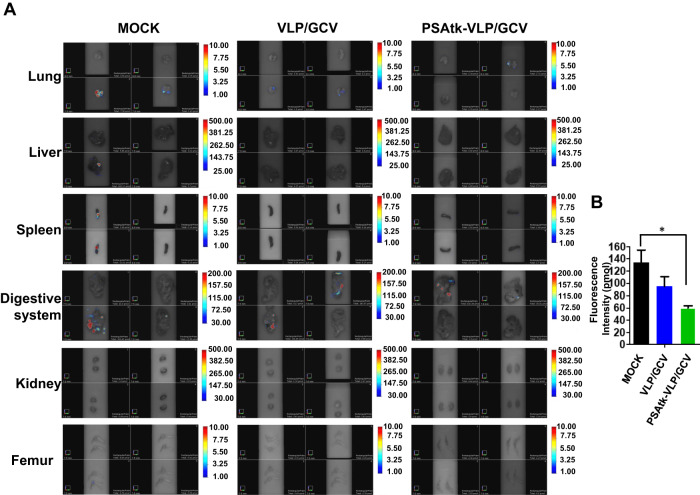
Organ fluorescence intensity detection at the end of the experiment (week 8) using fluorescence molecular tomography (FMT). 22Rv1-iRFP-GFP cells were implanted into mice anterior prostate lobes, and divided into three groups. The mock group received no treatment. The VLP/GCV and PSAtk-VLPs/GCV groups received seven cycles of virus-like particles (VLPs) and ganciclovir (GCV) or PSAtk-VLPs and GCV treatment, respectively. **A** FMT detection of the fluorescence intensity of the lung, liver, spleen, intestines, kidney, and femur in each mouse in all groups. **B** The fluorescence intensity of the metastatic tumor was obtained by ex vivo FMT imaging. *n* = 4, One-way ANOVA, *p < 0.05.

**Fig. 5:**
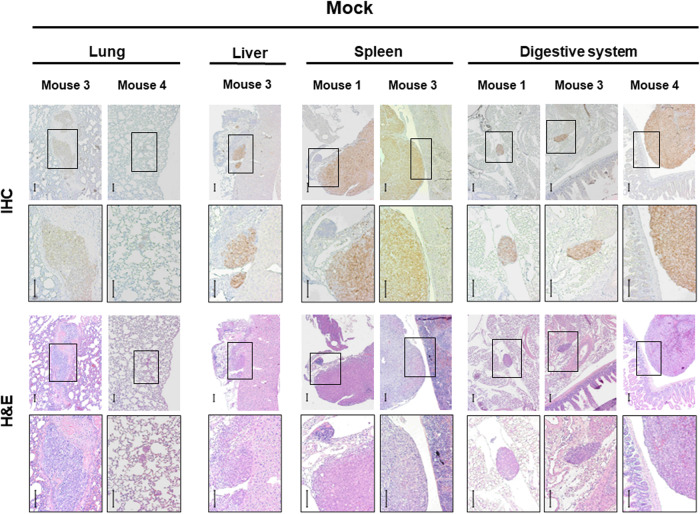
Immunohistochemistry (IHC) staining detection of androgen receptor (AR) expression in various organs of mice in the mock group. The organs of lungs, liver, spleen, and digestive system in the mock group were removed and stained with IHC for androgen receptor (AR), and the histological morphology was observed with hematoxylin and eosin (H&E) staining. IHC and H&E: the images are magnified image of the insert in the top row. Upper row scale bar = 100 μm (40× magnification), lower row (magnified image) scale bar = 100 μm (100× magnification). The metastatic tumor nodule in the digestive system of mouse 1 is within the mesenteric fatty tissue and shows mucosal villi in the small intestine for mice 3 and 4.

**Fig. 6:**
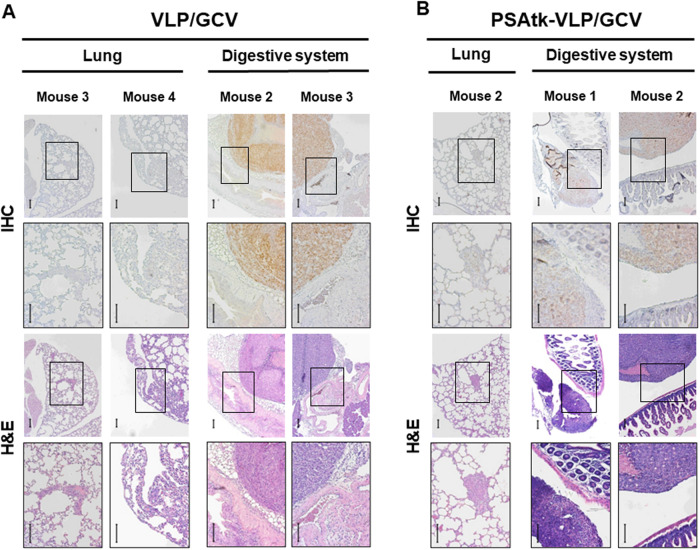
Immunohistochemistry (IHC) staining of androgen receptor (AR) expression in various organs of mice in the virus-like particle (VLP)/ganciclovir (GCV) and PSAtk-VLPs/GCV groups. At the end of the experiment, the lung and digestive system from **A** VLP/GCV and **B** PSAtk-VLPs/GCV mice were removed and stained with IHC for AR, and the histological morphology was observed with hematoxylin and eosin (H&E) staining. IHC and H&E: the images are magnified image of the insert in the top row. Upper row scale bar = 100 μm (40× magnification), lower row (magnified image) scale bar = 100 μm (100× magnification). **A** The metastatic tumor nodule in the digestive system of mouse 2 adheres peri-gallbladder fatty tissue, and adjacent to the peri-pancreatic vascular tissue in mouse 3. **B** The metastatic tumor nodule in the digestive system of mouse 1 situated next to small intestine (superior half), and neighbors to small intestine (inferior half) in mouse 2.

A challenge in treating prostate cancer is tumor metastasis [14]. FMT can detect fluorescence expression in deep tissues. In our assessment process of the therapeutic effects of PSAtk-VLP/GCV, apart from detecting fluorescence in orthotopic prostate cancer, the control group also exhibited more fluorescence signals in other organs, indicating a metastasis of tumor originating from orthotopic 22Rv1-iRFP-GFP tumor nodules; while the PSAtk-VLP/GCV group had weaker fluorescence signals in other organs (Suppl. Fig. 1, week 8, yellow arrow). We posit that PSAtk-VLP/GCV treatment can inhibit orthotopic prostate cancer metastasis. After week 8, the main organs, including lungs, liver, spleen, intestines, kidneys, and femur, were removed to detect fluorescence intensity with FMT and confirm tumor growth from the 22Rv1-iRFP-GFP metastasis using IHC staining. As shown in Fig. 4A and Table 1, the mock group exhibited fluorescence signals in organs such as the lungs, liver, spleen, and digestive system, excluding the kidneys and femur. VLP/GCV and PSAtk-VLP/GCV groups only detected signals in the lung and digestive tissue. Furthermore, fewer mice exhibited fluorescence in the PSAtk-VLP/GCV treatment group (Fig. 4A). FMT detection also indicated that after PSAtk-VLP/GCV treatment, the total fluorescence representing tumors also decreased (Fig. 4B). Therefore, PSAtk-VLP/GCV treatment inhibited orthotopic prostate cancer metastasis. IHC AR and H&E staining was used to confirm whether the tumors in various organs originated from the AR-expressing 22Rv1-iRFP-GFP tumor metastasis. The results indicated that AR expression could be detected in tumors of the lung, liver, spleen, and digestive system, in the mock group (Fig. 5). Similarly, in the VLP (Fig. 6A) and PSAtk-VLP/GCV (Fig. 6B) groups, AR expression could be detected, but fewer metastatic organs were detected in the PSAtk-VLP/GCV group (Fig. 6B, Table 1). In summary, systemic PSAtk-VLP delivery along with GCV treatment can effectively inhibit orthotopic prostate cancer metastasis to other organs.

**Table 1:** Metastasis profile of orthotopic prostate cancer murine models.

Groups	Local Growth	Lung Mets*	Liver Mets	Spleen Mets	Digestive system Mets	Kidney Mets	Femur Mets
MOCK *n* = 4	4/4 (100%)	2/4 (50%)	1/4 (25%)	2/4 (50%)	3/4 (75%)	0/4 (0%)	0/4 (0%)
VLP/GCV *n* = 4	4/4 (100%)	2/4 (50%)	0/4 (0%)	0/4 (0%)	2/4 (50%)	0/4 (0%)	0/4 (0%)
PSAtk-VLP/GCV *n* = 4	4/4 (100%)	1/4 (25%)	0/4 (0%)	0/4 (0%)	2/4 (50%)	0/4 (0%)	0/4 (0%)

22Rv1-iRFP-tGFP cancer cells were implanted using orthotopic routes. Columns represent number of mice with local growth at orthotopic site, or metastases at necropsy. (*Mets: Metastasis).

## Discussion

Despite new-generation drug development, drug resistance, and tumor metastasis in PCa patients still renders mCRP a lethal disease. Building on our previous findings, JCPyV VLPs encapsulating PSA promoter-driven tk suicide gene pPSAtk (PSAtk-VLPs) specifically inhibited the growth of the xenotransplantation of human CRPC cells into subcutaneous and bone metastatic prostate cancer in mice. In this study, we further explored the ability of PSAtk-VLPs to kill mCRPC orthotopic tumors and inhibit metastasis. The GFP gene encapsulated by JCPyV VLPs was specifically expressed in the mCRPC murine orthotopic tumor model we established. Using FMT imaging, we observed real-time growth and metastatic characteristics of human mCRPC orthotopic prostate cancer. After stably expressing the near-infrared fluorescent protein in mCRPC cells and orthotopically injecting them into the murine prostate, we systematically administered PSAtk-VLPs and GCV and observed that PSAtk-VLPs inhibited mCRPC orthotopic tumor growth and simultaneously inhibited mCRPC tumor metastasis to other organs.

The majority of prostate cancer patients die from metastatic diseases [14], which strongly underscores the necessity to devise a strategy for a systemic treatment to inhibit mCRPC metastasis. It is particularly important to establish appropriate animal models to provide appropriate information for preclinical research. Previously, the near-infrared protein was applied to orthotopic prostate cancer murine models to evaluate the microenvironment of microvessels and how it enables prostate cancer metastasis [33]. Further, it has also been applied in evaluations during surgery to differentiate the boundaries between tumor and normal tissue, guiding the range of surgical boundaries [34]. Given that near-infrared fluorescent wavelengths are longer and capable of penetrating deep tissues, coupled with their low background values in tissue, they are particularly well-suited for research on tumor metastasis [35]. Therefore, taking advantage of such characteristics, we established an orthotopic cancer model with mCRPC 22Rv1 tumors carrying near-infrared proteins. We used FMT to detect the fluorescence signals from organ locations representing mCRPC 22Rv1 metastasis weekly, and these signals were consistent with IHC staining-determined AR expression in different organ locations. This further indicates that our mCRPC 22Rv1 orthotopic cancer model reflects the positions of metastatic tumors.

In clinical settings, mCRPC patients often develop metastasis to the bone and lymph nodes, and nearly 20% of patients develop multi-site organ metastases, such as in the liver and lung and suffer from low survival rates [36, 37]. Here, we did not detect any signals in the bone location via FMT fluorescence or IHC staining in the orthotopic prostate cancer mouse model we established. However, in organs such as the liver and lung, where patients often experience metastasis, we were able to detect prostate tumor metastasis. Tumor metastasis usually involves interactions between the tumor and its microenvironment [38]. In this human orthotopic prostate cancer murine model, the lack of bone metastasis necessitates further investigation to determine if variations in the tumor microenvironment could be responsible.

Presently, numerous strategies for prostate cancer gene therapy have been developed. These strategies include tumor suppressor gene activation, direct tumor growth inhibition, prodrug toxicity, anti-tumor vaccine activation, and tumor microenvironment alteration [17]. Vector specificity significantly influences the gene therapy efficacy. JCPyV can infect prostate cells and is associated with human prostate cancer development, which implies that prostate cancer cells are permissive to JCPyV. Furthermore, prostate cancer progression vitally involves PSA. Therefore, we encapsulated the therapeutic tk gene driven by the PSA promoter within the VLP derived from JCPyV. In the presence of the tk gene, the prodrug GCV can be converted into toxic ganciclovir triphosphate which exhibits a bystander effect and a strong cytotoxic effect. As a result, we observed a reduction in the size and quantity of tumors—whether orthotopic prostate cancer or metastasized tumors in other organs—post-treatment with PSAtk-VLP carried by JCPyV VLP. Our findings suggest the potential of JCPyV VLP as a gene therapy vector that can protect and deliver genes to specific organs via systemic gene delivery. The tumor-microenvironment interaction regulates tumor metastasis. In the future, the specificity of JCPyV VLP can be used to design targeting factors associated with tumor metastasis interaction to assess the ability of JCPyV VLP to inhibit tumor cell metastasis.

It is challenging for systemic therapy to track and target metastatic tumors with toxicity without harming other cells. Previously, we used a PSA promoter to drive the tk suicide gene. In the presence of the prodrug, GCV, JCPyV VLP can selectively target prostate tumors, leaving bladder tumors without PSA unaffected [21]. This study offers further confirmation that JCPyV VLPs delivering genes to prostate tumors are not influenced by the tumor microenvironment and can also inhibit orthotopic tumor metastasis. Therefore, when combined with tumor-specific markers, JCPyV VLPs present potential as specific carriers for various tumor treatments. Most people have been infected by JCPyV. The seropositive status may cause decreased efficiency due to immune elimination when using the JCPyV VLP as a gene delivery vector. Modification of the JCPyV VLPs may be a strategy for using the JCPyV VLP as a gene delivery vector in the future [39].

In conclusion, our study demonstrates JCPyV VLPs as gene carriers for systemic prostate cancer therapy. In the future, the prospect of gene therapy, facilitated by the delivery of therapeutic genes via JCPyV VLPs, could present an additional treatment option for patients with prostate cancer.

### Supplementary information

Supplementary Fig. 1

Supplementary Fig. 2

## Data Availability

The datasets used and analyzed during the current study are available from the corresponding author upon reasonable request.
