# Supplementary material for: Inhibition of orthotopic castration-resistant prostate cancer growth and metastasis in mice by JC VLPs carrying a suicide gene driven by the PSA promoter
Source: Cancer Gene Ther. 2023 Dec 11;31(2):250–8. doi: 10.1038/s41417-023-00699-8 (PMC10874888; doi:10.1038/s41417-023-00699-8)

Supplementary Fig. 1 Fluorescence molecular tomography (FMT) imaging monitoring the whole-body fluorescence intensity of each mouse per group on a weekly basis. 22Rv1-iRFP-GFP cells were implanted into the anterior prostate lobe of mice and divided into three groups, the mock group, the VLP/GCV, and PSAtk-VLPs/GCV groups. Each week, the fluorescence intensity representing tumor growth was traced and detected using FMT. Scale bar = 100 μm. (White arrows indicate detected fluorescence; Yellow arrows indicate metastasis tumor fluorescence).

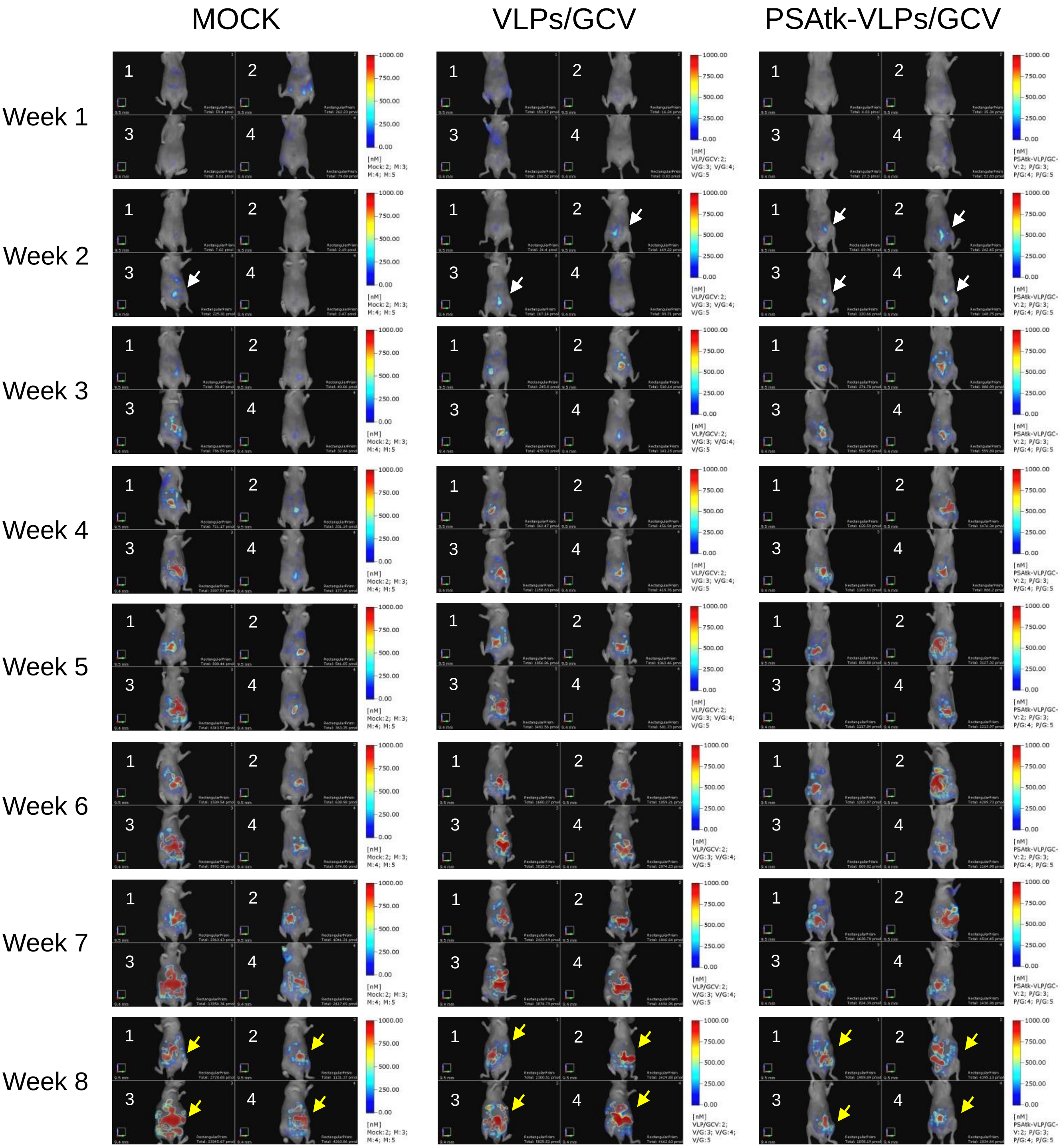

Supplement: Supplementary file 1 — Supplementary Fig. 1 [file 41417_2023_699_MOESM1_ESM.pdf]
