# Supplementary material for: Inhibition of orthotopic castration-resistant prostate cancer growth and metastasis in mice by JC VLPs carrying a suicide gene driven by the PSA promoter
Source: Cancer Gene Ther. 2023 Dec 11;31(2):250–8. doi: 10.1038/s41417-023-00699-8 (PMC10874888; doi:10.1038/s41417-023-00699-8)

Supplementary Fig. 2 Immunohistochemistry (IHC) staining detection of PSA and Ki-67 expression and hematoxylin and eosin (H&E) staining in orthotopic prostate tumors. Scale bar = 100 μm

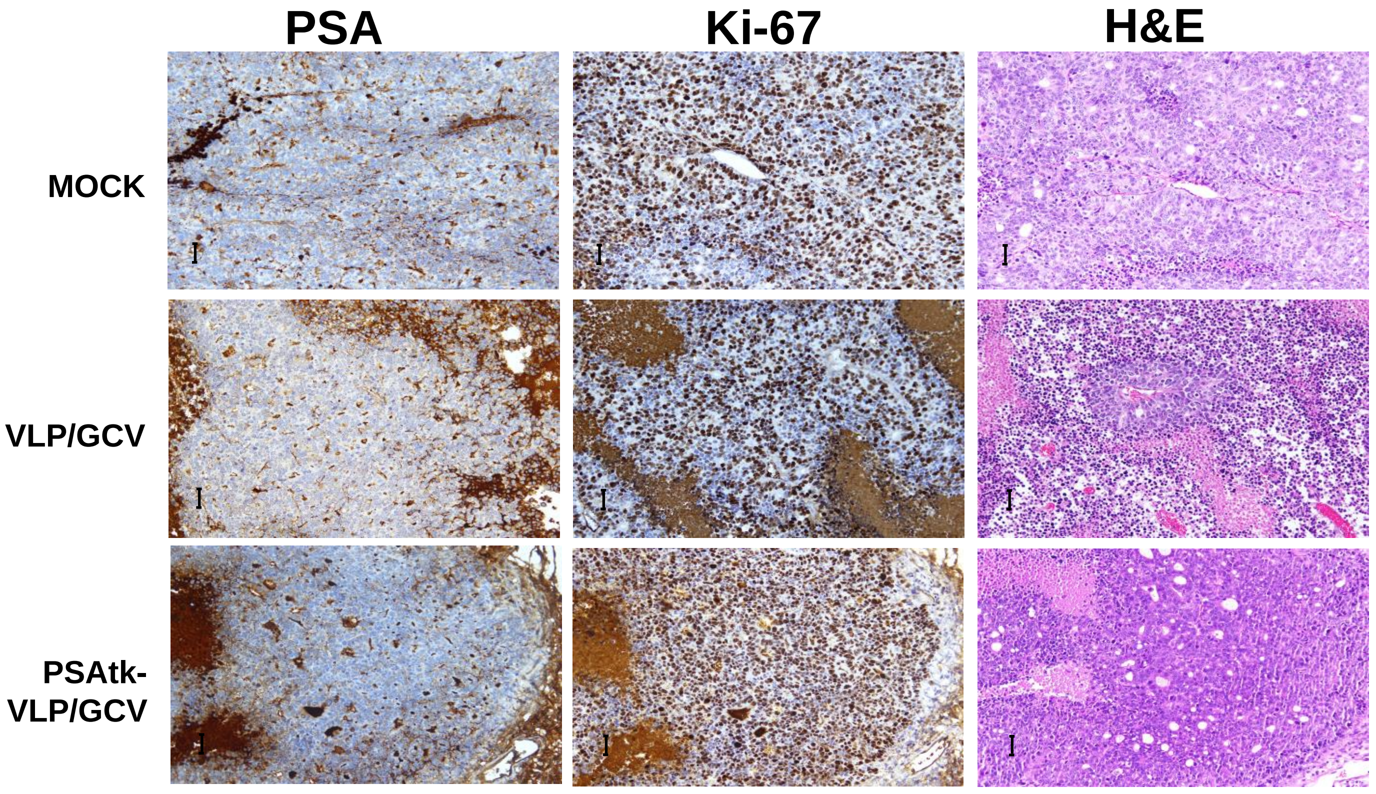

Supplement: Supplementary file 2 — Supplementary Fig. 2 [file 41417_2023_699_MOESM2_ESM.pdf]
